# Supplementary material for: Time trends between 2002 and 2017 in correlates of self-reported sitting time in European adults
Source: PLoS One. 2019 Nov 12;14(11):e0225228. doi: 10.1371/journal.pone.0225228 (PMC6850696; doi:10.1371/journal.pone.0225228)
Supplement: S1 Table — (DOCX) [file pone.0225228.s001.docx]

S1 Table. Total population, high sitters and high sitters-least active by country for all four surveys.

| \|  \| **2002** \| \| \| \| **2005** \| \| \| \| **2013** \| \| \| \| **2017** \| \| \| \| \| --- \| --- \| --- \| --- \| --- \| --- \| --- \| --- \| --- \| --- \| --- \| --- \| --- \| --- \| --- \| --- \| --- \| \|  \| **% European population** \| **% total population based on internal country weights** \| **% sitting >7.5 h/d** \| **N % high sit/low active *** \| **% European population** \| **% total population based on internal country weights** \| **% sitting >7.5 h/d** \| **N % high sit/low active *** \| **% European population** \| **% total population based on internal country weights** \| **% sitting >7.5 h/d** \| **N % high sit/low active *** \| **% European population** \| **% total population based on internal country weights** \| **% sitting >7.5 h/d** \| **N % high sit/low active *** \| \| \| **Northern Europe** \| 22.0 \|  \| 22.2 \| 11.3 \| 15.8 \|  \| 24.0 \| 9.8 \| 18.8 \|  \| 21.1 \| 8.4 \| 18.5 \|  \| 20.8 \| 7.5 \| \| Denmark \| 1.4 \| 6.4 \| 36.3 \| 10.5 \| 1.0 \| 3.6 \| 34.5 \| 11.7 \| 1.1 \| 3.7 \| 33.0 \| 11.6 \| 1.1 \| 3.6 \| 33.2 \| 11.5 \| \| Estonia \|  \|  \|  \|  \| 0.2 \| 3.4 \| 24.0 \| 7.0 \| 0.2 \| 3.7 \| 22.9 \| 9.4 \| 0.3 \| 3.5 \| 25.1 \| 10.3 \| \| Finland \| 1.4 \| 6.5 \| 31.8 \| 9.9 \| 1.0 \| 3.6 \| 30.5 \| 11.0 \| 1.1 \| 3.5 \| 24.8 \| 7.1 \| 1.1 \| 3.7 \| 18.7 \| 6.9 \| \| Ireland \| 1.0 \| 6.3 \| 16.7 \| 7.2 \| 0.7 \| 3.3 \| 16.5 \| 8.4 \| 0.8 \| 3.6 \| 10.7 \| 5.0 \| 0.8 \| 3.6 \| 16.3 \| 7.4 \| \| Latvia \|  \|  \|  \|  \| 0.3 \| 3.5 \| 19.3 \| 7.1 \| 0.4 \| 3.6 \| 17.5 \| 5.8 \| 0.4 \| 3.6 \| 21.1 \| 8.8 \| \| Lithuania \|  \|  \|  \|  \| 0.6 \| 3.1 \| 18.2 \| 5.6 \| 0.7 \| 3.7 \| 16.4 \| 7.9 \| 0.6 \| 3.6 \| 18.2 \| 9.6 \| \| Sweden \| 2.4 \| 6.4 \| 29.6 \| 12.8 \| 1.7 \| 3.7 \| 27.4 \| 11.2 \| 1.9 \| 3.6 \| 28.7 \| 6.4 \| 1.9 \| 3.9 \| 31.5 \| 7.9 \| \| UK \| 15.8 \| 8.4 \| 19.3 \| 12.1 \| 10.4 \| 4.3 \| 22.7 \| 10.6 \| 12.6 \| 4.8 \| 19.6 \| 10.0 \| 12.2 \| 4.8 \| 18.5 \| 7.8 \| \| **Western Europe** \| 46.3 \|  \| 23.4 \| 9.8 \| 31.2 \|  \| 23.0 \| 8.0 \| 34.7 \|  \| 20.1 \| 7.4 \| 36.4 \|  \| 20.4 \| 8.1 \| \| Austria \| 1.9 \| 5.7 \| 22.7 \| 8.0 \| 1.6 \| 3.7 \| 17.4 \| 8.0 \| 1.7 \| 3.6 \| 19.1 \| 7.1 \| 1.7 \| 3.6 \| 17.3 \| 8.0 \| \| Belgium \| 2.8 \| 6.7 \| 24.2 \| 12.9 \| 2.1 \| 3.6 \| 26.4 \| 12.1 \| 2.2 \| 3.9 \| 19.3 \| 10.2 \| 2.3 \| 3.7 \| 21.4 \| 10.7 \| \| France \| 15.8 \| 6.5 \| 18.6 \| 10.8 \| 10.4 \| 3.6 \| 16.7 \| 7.7 \| 11.9 \| 3.7 \| 18.7 \| 8.8 \| 12.7 \| 3.7 \| 16.7 \| 8.7 \| \| Germany \| 21.4 \| 12.1 \| 26.3 \| 10.4 \| 10.3 \| 5.1 \| 23.9 \| 7.1 \| 15.6 \| 5.7 \| 18.6 \| 6.1 \| 16.4 \| 5.7 \| 19.4 \| 7.1 \| \| Luxembourg \| 0.1 \| 3.7 \| 25.5 \| 9.2 \| 0.1 \| 1.8 \| 24.3 \| 11.4 \| 0.1 \| 1.8 \| 23.7 \| 8.1 \| 0.1 \| 1.7 \| 18.7 \| 7.4 \| \| Netherlands \| 4.2 \| 6.4 \| 26.9 \| 5.7 \| 3.1 \| 3.6 \| 40.9 \| 6.5 \| 3.2 \| 3.7 \| 33.4 \| 10.8 \| 3.1 \| 3.6 \| 41.4 \| 12.4 \| \| **Eastern Europe** \|  \|  \|  \|  \| 17.8 \|  \| 22.7 \| 7.1 \| 18.5 \|  \| 17.9 \| 8.7 \| 17.7 \|  \| 19.9 \| 9.9 \| \| Bulgaria \|  \|  \|  \|  \| 1.5 \| 3.4 \| 18.5 \| 5.1 \| 1.5 \| 3.6 \| 19.4 \| 9.9 \| 1.4 \| 3.5 \| 22.0 \| 10.2 \| \| Czech Republic \|  \|  \|  \|  \| 1.9 \| 3.3 \| 36.1 \| 12.5 \| 2.2 \| 3.7 \| 26.0 \| 11.6 \| 2.1 \| 3.7 \| 29.0 \| 16.1 \| \| Hungary \|  \|  \|  \|  \| 2.0 \| 3.5 \| 16.6 \| 6.5 \| 2.0 \| 3.7 \| 11.5 \| 6.4 \| 2.0 \| 3.7 \| 17.2 \| 9.8 \| \| Poland \|  \|  \|  \|  \| 7.2 \| 3.4 \| 27.3 \| 9.3 \| 7.3 \| 3.3 \| 18.2 \| 10.1 \| 7.3 \| 3.4 \| 20.2 \| 11.0 \| \| Romania \|  \|  \|  \|  \| 4.2 \| 3.5 \| 12.9 \| 2.8 \| 4.2 \| 3.5 \| 14.9 \| 6.4 \| 3.7 \| 3.4 \| 15.4 \| 5.3 \| \| Slovakia \|  \|  \|  \|  \| 1.0 \| 3.6 \| 23.8 \| 5.8 \| 1.1 \| 3.6 \| 20.4 \| 7.6 \| 1.0 \| 3.7 \| 17.6 \| 9.6 \| \| **Southern Europe** \| 31.7 \|  \| 21.3 \| 9.6 \| 35.2 \|  \| 17.2 \| 6.5 \| 28.0 \|  \| 11.5 \| 5.1 \| 27.5 \|  \| 16.0 \| 9.5 \| \| Croatia \|  \|  \|  \|  \| 0.9 \| 3.6 \| 24.8 \| 9.6 \| 0.9 \| 3.7 \| 22.8 \| 8.4 \| 0.9 \| 3.8 \| 17.7 \| 10.3 \| \| Cypress-TCC \|  \|  \|  \|  \| 13.9 \| 1.5 \| 22.9 \| 13.3 \|  \|  \|  \|  \|  \|  \|  \|  \| \| Cyprus \|  \|  \|  \|  \| 0.1 \| 1.7 \| 33.6 \| 18.7 \| 0.2 \| 1.8 \| 19.6 \| 13.5 \| 0.2 \| 1.8 \| 20.7 \| 15.3 \| \| Greece \| 2.9 \| 6.5 \| 22.3 \| 8.4 \| 2.1 \| 3.7 \| 34.3 \| 11.0 \| 2.1 \| 3.7 \| 20.3 \| 10.1 \| 2.3 \| 3.6 \| 24.9 \| 15.0 \| \| Italy \| 16.1 \| 6.4 \| 24.3 \| 11.8 \| 11.7 \| 3.6 \| 14.1 \| 6.2 \| 12.5 \| 3.6 \| 11.0 \| 5.2 \| 12.2 \| 3.7 \| 16.4 \| 10.7 \| \| Malta \|  \|  \|  \|  \| 0.1 \| 1.6 \| 12.7 \| 6.5 \| 0.1 \| 1.8 \| 15.3 \| 7.4 \| 0.1 \| 1.8 \| 21.1 \| 12.6 \| \| Portugal \| 2.6 \| 6.1 \| 11.8 \| 4.0 \| 1.9 \| 3.6 \| 10.4 \| 3.7 \| 2.0 \| 3.7 \| 10.0 \| 6.2 \| 1.9 \| 3.8 \| 16.5 \| 13.7 \|   Notes:  Abbreviations: h/d: hours per day; na: not applicable; PA: physical activity  *High sit/low active = sitting more than 7.5 hours per day and being in the least active PA quartile |
| --- | --- | --- | --- | --- | --- | --- | --- | --- | --- | --- | --- | --- | --- | --- | --- | --- | --- | --- | --- | --- | --- | --- | --- | --- | --- | --- | --- | --- | --- | --- | --- | --- | --- | --- | --- | --- | --- | --- | --- | --- | --- | --- | --- | --- | --- | --- | --- | --- | --- | --- | --- | --- | --- | --- | --- | --- | --- | --- | --- | --- | --- | --- | --- | --- | --- | --- | --- | --- | --- | --- | --- | --- | --- | --- | --- | --- | --- | --- | --- | --- | --- | --- | --- | --- | --- | --- | --- | --- | --- | --- | --- | --- | --- | --- | --- | --- | --- | --- | --- | --- | --- | --- | --- | --- | --- | --- | --- | --- | --- | --- | --- | --- | --- | --- | --- | --- | --- | --- | --- | --- | --- | --- | --- | --- | --- | --- | --- | --- | --- | --- | --- | --- | --- | --- | --- | --- | --- | --- | --- | --- | --- | --- | --- | --- | --- | --- | --- | --- | --- | --- | --- | --- | --- | --- | --- | --- | --- | --- | --- | --- | --- | --- | --- | --- | --- | --- | --- | --- | --- | --- | --- | --- | --- | --- | --- | --- | --- | --- | --- | --- | --- | --- | --- | --- | --- | --- | --- | --- | --- | --- | --- | --- | --- | --- | --- | --- | --- | --- | --- | --- | --- | --- | --- | --- | --- | --- | --- | --- | --- | --- | --- | --- | --- | --- | --- | --- | --- | --- | --- | --- | --- | --- | --- | --- | --- | --- | --- | --- | --- | --- | --- | --- | --- | --- | --- | --- | --- | --- | --- | --- | --- | --- | --- | --- | --- | --- | --- | --- | --- | --- | --- | --- | --- | --- | --- | --- | --- | --- | --- | --- | --- | --- | --- | --- | --- | --- | --- | --- | --- | --- | --- | --- | --- | --- | --- | --- | --- | --- | --- | --- | --- | --- | --- | --- | --- | --- | --- | --- | --- | --- | --- | --- | --- | --- | --- | --- | --- | --- | --- | --- | --- | --- | --- | --- | --- | --- | --- | --- | --- | --- | --- | --- | --- | --- | --- | --- | --- | --- | --- | --- | --- | --- | --- | --- | --- | --- | --- | --- | --- | --- | --- | --- | --- | --- | --- | --- | --- | --- | --- | --- | --- | --- | --- | --- | --- | --- | --- | --- | --- | --- | --- | --- | --- | --- | --- | --- | --- | --- | --- | --- | --- | --- | --- | --- | --- | --- | --- | --- | --- | --- | --- | --- | --- | --- | --- | --- | --- | --- | --- | --- | --- | --- | --- | --- | --- | --- | --- | --- | --- | --- | --- | --- | --- | --- | --- | --- | --- | --- | --- | --- | --- | --- | --- | --- | --- | --- | --- | --- | --- | --- | --- | --- | --- | --- | --- | --- | --- | --- | --- | --- | --- | --- | --- | --- | --- | --- | --- | --- | --- | --- | --- | --- | --- | --- | --- | --- | --- | --- | --- | --- | --- | --- | --- | --- | --- | --- | --- | --- | --- | --- | --- | --- | --- | --- | --- | --- | --- | --- | --- | --- | --- | --- | --- | --- | --- | --- | --- | --- | --- | --- | --- | --- | --- | --- | --- | --- | --- | --- | --- | --- | --- | --- | --- | --- | --- | --- | --- | --- | --- | --- | --- | --- | --- | --- | --- | --- | --- | --- | --- | --- | --- | --- | --- | --- | --- | --- | --- | --- | --- | --- | --- | --- | --- | --- | --- | --- | --- | --- | --- | --- | --- | --- | --- | --- | --- | --- | --- | --- | --- | --- | --- | --- | --- | --- | --- | --- | --- | --- | --- | --- | --- | --- | --- | --- | --- | --- | --- | --- | --- | --- | --- | --- | --- | --- | --- | --- | --- | --- | --- | --- | --- |
